# Supplementary material for: Read my LIPSS: organic lasers on micromachined resonators
Source: Nat Commun. 2025 Aug 1;16:7057. doi: 10.1038/s41467-025-62502-6 (PMC12316998; doi:10.1038/s41467-025-62502-6)
Supplement: Supplementary file 1 — Supplementary Information [file 41467_2025_62502_MOESM1_ESM.pdf]

Read my LIPSS: organic lasers on micromachined resonators

Tiange Dong[*,1,2], Tobias Antrack[*,1,2], Frithjof Pietsch[2], Jakob Lindenthal[2], Markus Löffler[3], Bernd Rellinghaus[3], Johannes Benduhn[2], Markas Sudzius[2], and Karl Leo[*,2]*

[1] Authors contributed equally to this work

[2] Dresden Integrated Center for Applied Physics and Photonic Materials (IAPP) and Institute of Applied Physics, TU Dresden, Nöthnitzer Str. 61, 01187 Dresden, Germany.

[3] Dresden Center for Nanoanalysis (DCN), Center for Advancing Electronics Dresden (CFAED), Technische Universität Dresden, 01069 Dresden, Germany

Email addresses: tiange.dong@mpi-halle.mpg.de, tobias.antrack@tu-dresden.de, karl.leo@tu-dresden.de

SUPPLEMENTARY INFORMATION

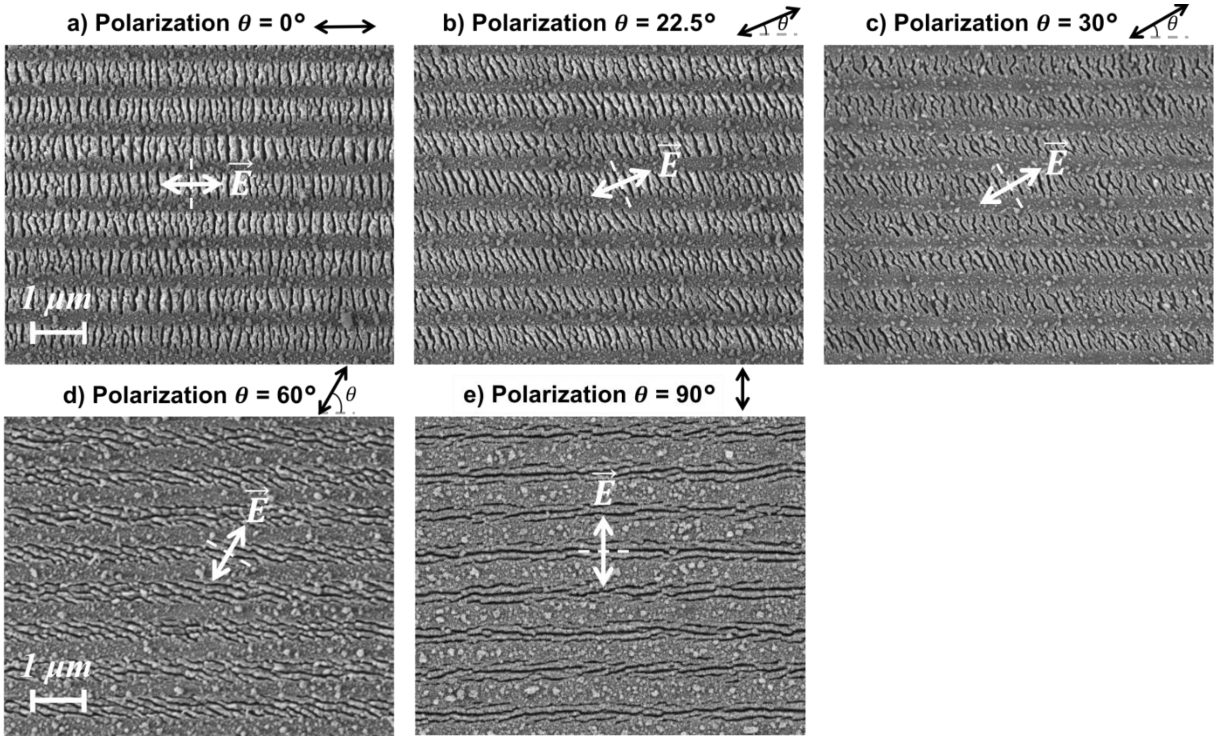

Supplementary Figure 1 | Characterization of the laser polarization dependency in the dynamic structuring approach. The applied laser polarization angles are a) θ = 0°, b) θ = 22.5°, c) θ = 30°, d) θ = 60°, and e) θ = 90°, represented by the inset arrow lines. The pulse fluences for a-c) were 82 nJ, and 78 nJ for d) and e). The SEM images of the resulting structures clearly show that the orientation of dynamic-scanned laser-induced nano-gratings is perpendicular to incoming laser polarization.

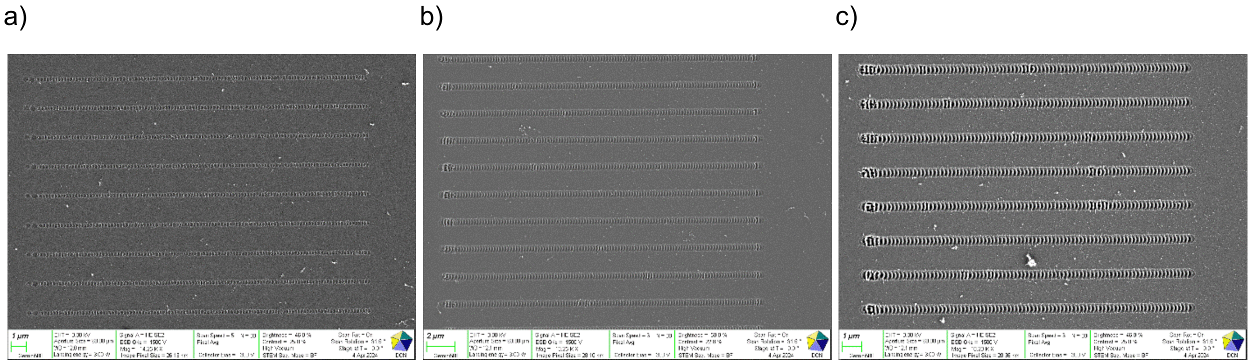

Supplementary Figure 2 | LIPSS formation under different structuring laser fluence. All samples were fabricated with the same shot-to-shot spacing of 194 nm and the same speed of 1 mm/s while the fluence of the impinging laser beam was varied (a: 70 nJ, b: 86 nJ, c: 104 nJ). Fourier analysis of the SEM-scans reveals that the dominant periodicities are 193.90 nm for a, 193.11 nm for b, and 194.87 nm for c. The resulting widths of the LIPSS stripes are 287 nm for a, 478 nm for b, and 489 nm for c.

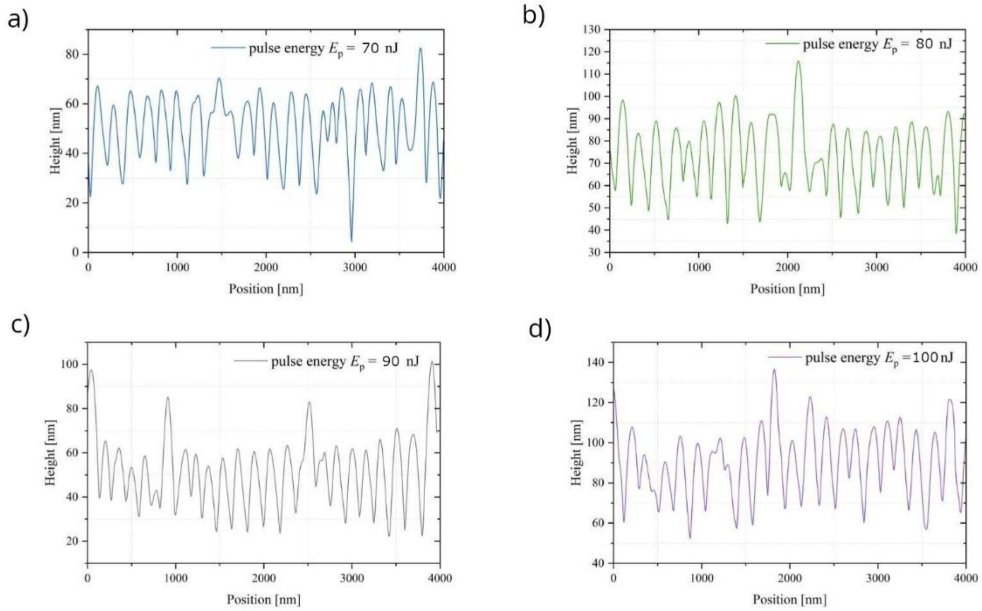

Supplementary Figure 3 | LIPSS grating amplitude. 4 samples were prepared with the same shot-to-shot spacing of 189 nm but with varying pulse fluence. The presented AFM height profile measurements reveal average depths of 32.5 nm for 70 nJ pulse fluence, 34.8 nm for 80 nJ, 36.0 nm for 90 nJ, and 38.6 nm for 100 nJ.

Supplementary Table 1 | Simulation parameters. The parameters were chosen to resemble the LIPSS gratings as realistic as possible. For different grating lengths, the reflectivity was determined by detecting the power of reflected monochromatic waves.

**Parameter**	**Value**
grating position	top and bottom of waveguide
grating profile	sinusoidal or<br>realistic profile from AFM data
grating amplitude	30 nm
periodicity	186 nm
waveguide height	500 nm
refractive index of waveguide	1.78
refractive index of substrate	1.458

a) b)

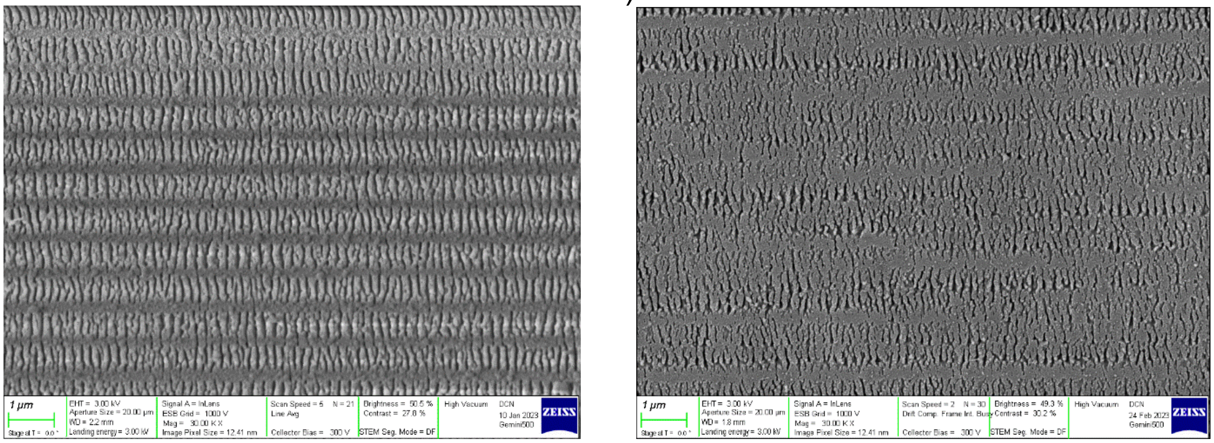

Supplementary Figure 4 | Instability of LIPSS with distance of adjacent lines below 700 nm. a LIPSS gratings produced with a line distance of 730 nm and **b** 644 nm. The close proximity of an already existing LIPSS grating to the impinging structuring laser beam during fabrication could influence the material self-organization processes.

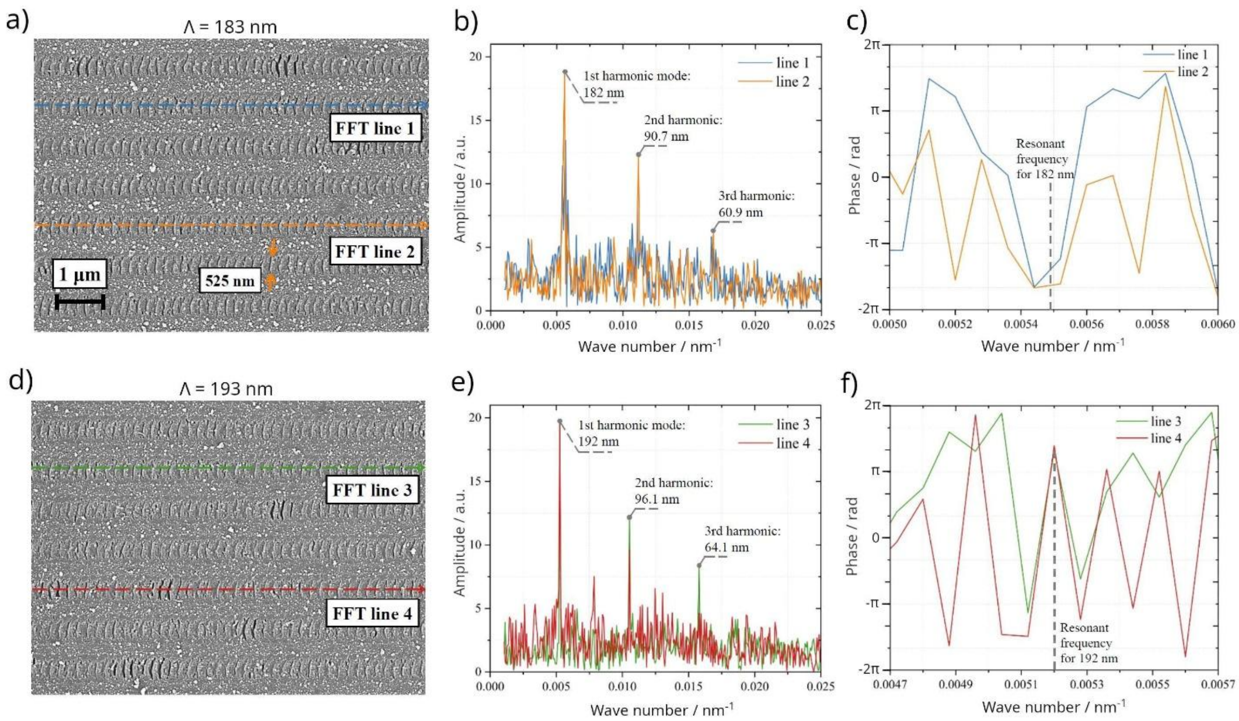

Supplementary Figure 5 | Surface morphology and fast Fourier transformation. The parallel-scanned first-order DFB resonators were fabricated with pulse distances of a) - c) 183 nm, and d) - f) 193 nm. Two analysis lines were selected from each sample and checked the amplitude and phase difference to evaluate errors. b) and e) give the dominant periodicity of 182 nm and 192 nm, and are identical between line 1 and 2, as well as line 3 and 4. c) and f) indicate the phase-locking between different raster scans with a phase difference of only 8° and 2° for the two samples. These supplemented data support our statement in the main text of high fabricating accuracy and reproducibility for every individual scan.

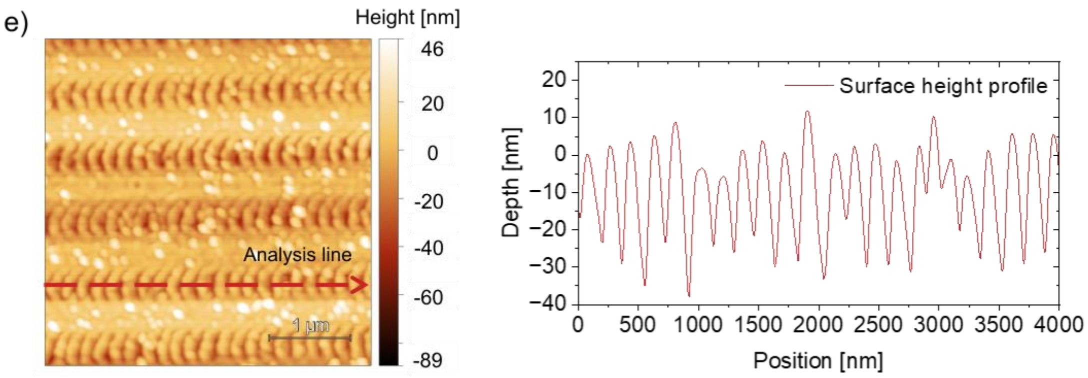

Supplementary Figure 6 | Atomic force microscope image of a LIPSS grating and the corresponding height profile. The similarity to the obtained SEM images (Supplementary Figure 5) is notable. Therefore, it is sufficient to only use the SEM images for the Fourier analysis.

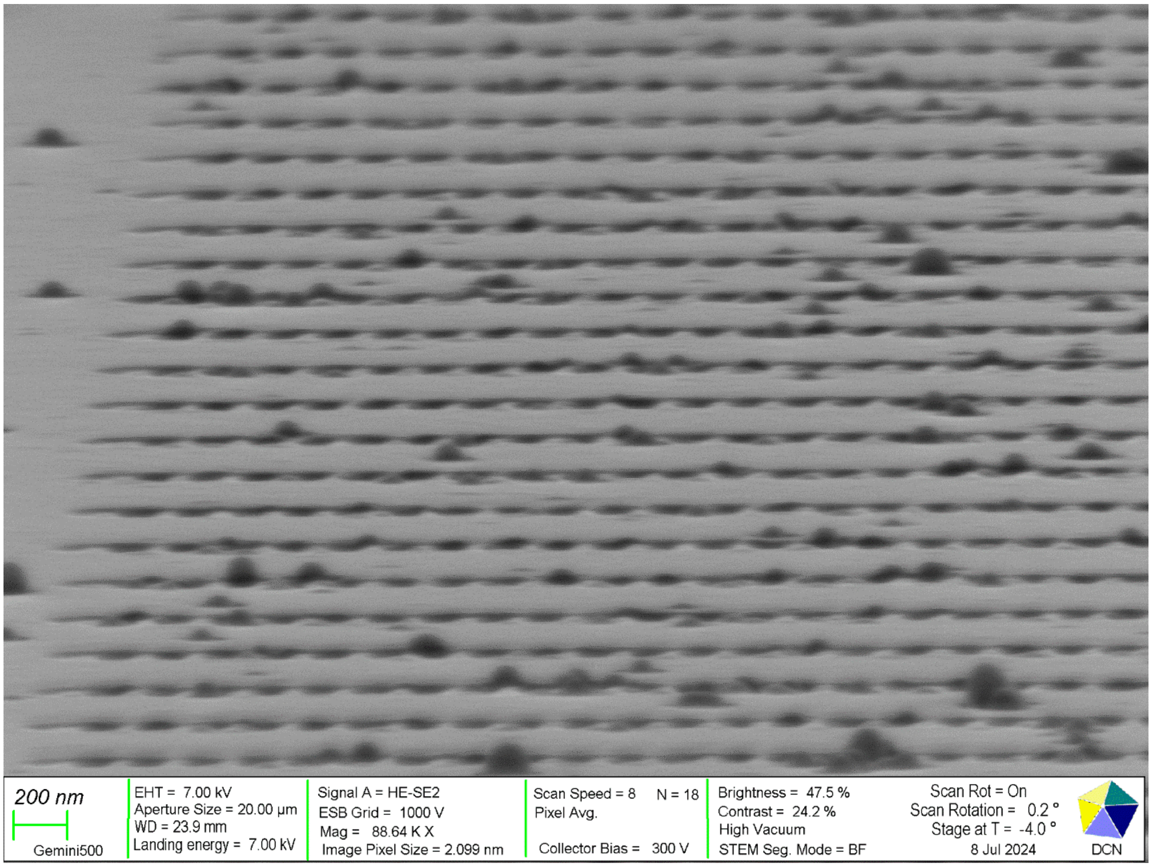

Supplementary Figure 7 | SEM image of the Alq_3:DCM top surface on top of multiple LIPSS lines. The sample is tilted with an angle of 4° and the surface was sputtered with 4 nm of carbon to avoid charging during the measurement. The distance between the lines is 1.6 µm. The phase stability of the LIPSS gratings is clearly visible here, which allows efficient coupling between single lines.

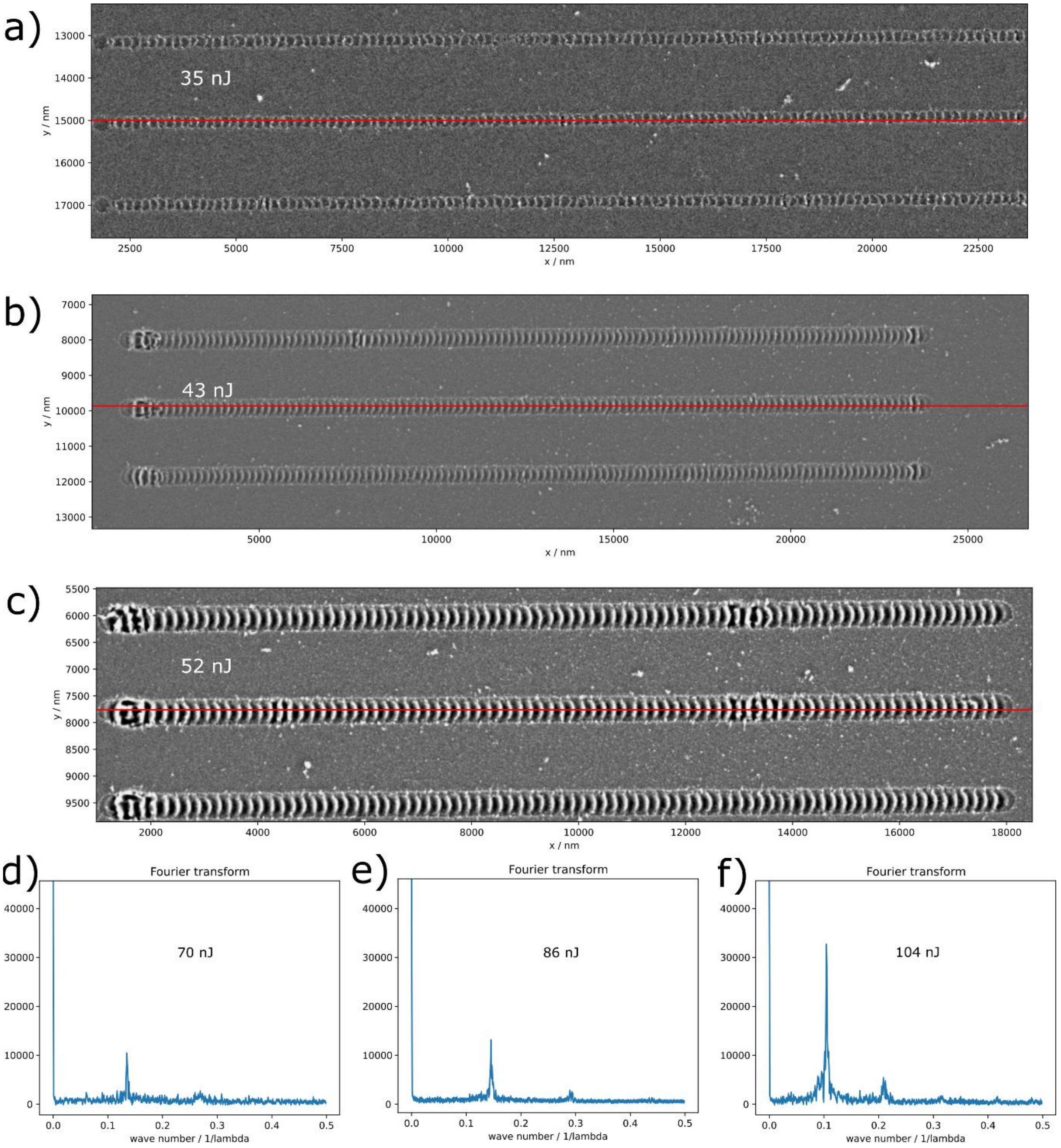

Supplementary Figure 8 | Fourier Analysis of different LIPSS gratings. The LIPSS structures were fabricated with different laser pulse fluence but the same pulse-to-pulse spacing of 194 nm. Subfigures a-c) show the LIPSS structures shown in Supplementary Figure 2 in more detail (LIPSS gratings with the same pulse-to-pulse spacing but varied laser pulse fluence. The red lines mark the horizontal pixel line of the image that was used for the Fourier Transformation. The resulting frequency intensities of the Fourier Transformation of the red lines are presented in d-f). The carrier periodicities (e.g. frequencies with the highest intensity) are 193.90 nm for d), 193.11 nm for d), and 194.87 nm for f).

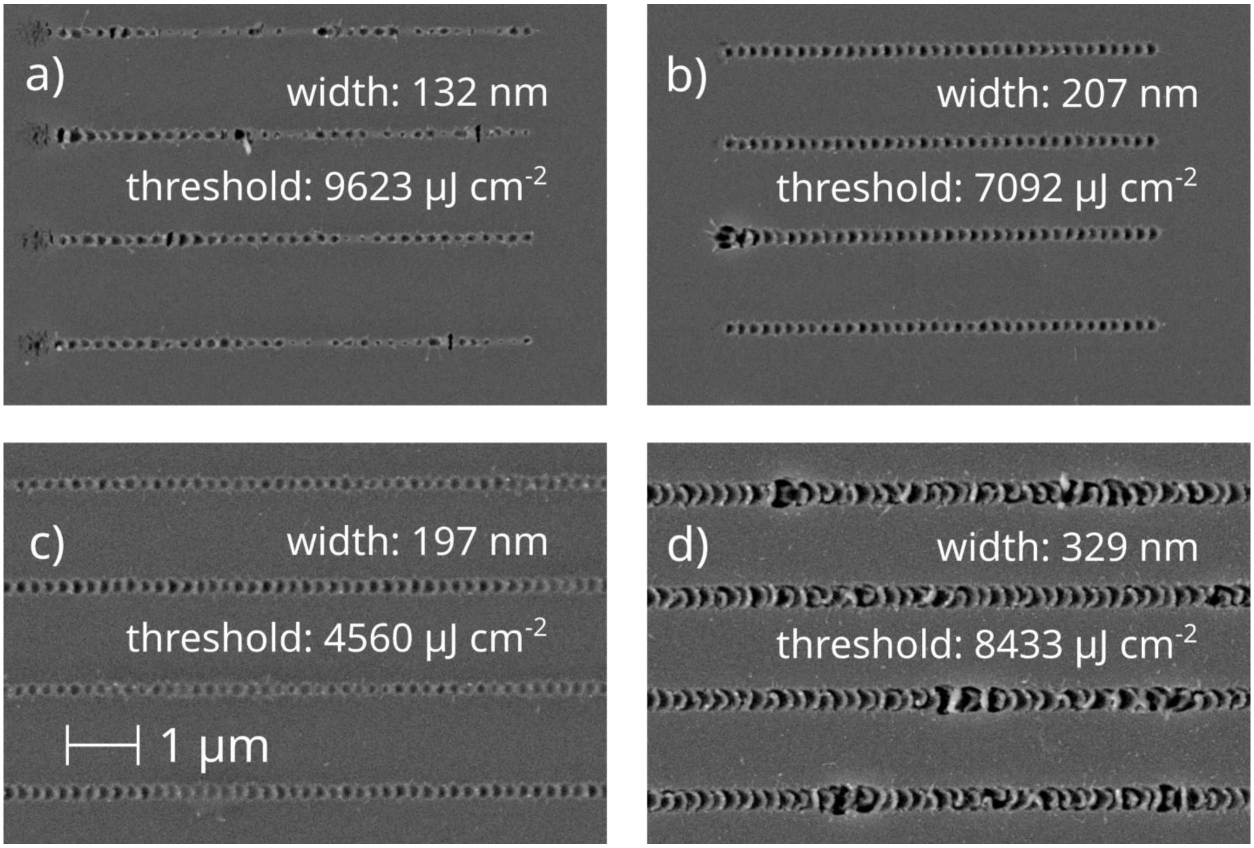

Supplementary Figure 9: Comparison of different lasing thresholds for different LIPSS stripe widths. Subfigure a) and b) show 6 µm long LIPSS stripes where the stripes in b) are significantly wider. Consequently, the measured lasing threshold is lower due to the larger volume which acts as a DFB reflector. The LIPSS lines in c) and d) have a length of 17 µm. Due to the curved appearance of the corrugation and the visible irregularities, the observed threshold is higher for d) although a larger area could serve as a DFB reflector. With increased fabrication power, the gratings are expected to become deeper and therefore enhance optical feedback However, this is only true for a certain range, because the waveguiding efficiency is negatively influenced by high grating amplitudes[1]. The lateral spacing between the LIPSS lines is 1.416 µm for all samples.

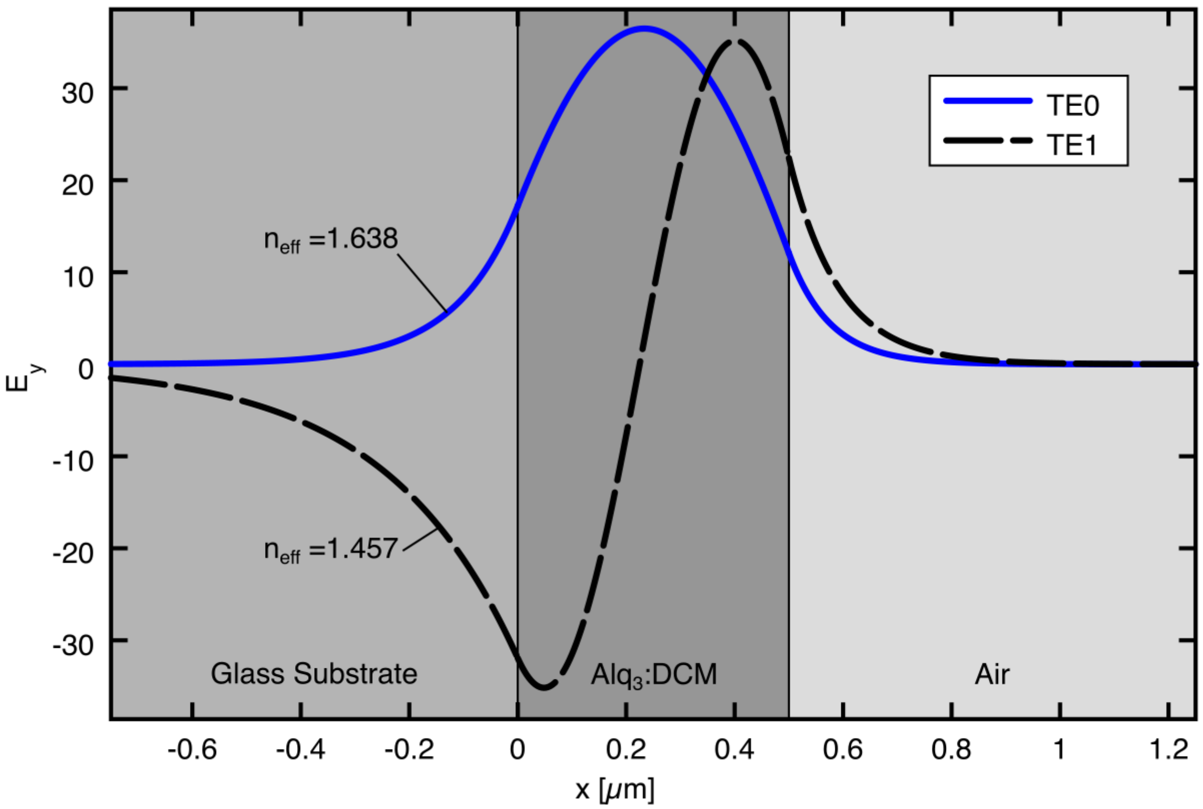

Supplementary Figure 10: Electric field distribution for the waveguided modes with a wavelength of 618 nm, where the resulting effective refractive index of the TE0 mode perfectly matches to the experimentally observed efffective refractive index of n_{eff} = 1.64. It can be clearly seen that the evanescent part of the waveguided TE0 mode reaches into the surrounding layers, with a higher field strength in the glass substrate. The assumed refractive indices are n = 1.4 for the glass substrate[2] (left side), n = 1.7 for the Alq_3:DCM[2] gain material (middle), and n = 1 for the air (rigth side). These results were obtained using the online 1D mode solver OMS (https://www.siio.eu/oms.html). With the field strength being higher on the glass/organic interface than on the organic/air interface, the actual LIPSS corrugation is expected to supply the optical feedback for lasing (rather than the corrugation on the top surface of the organic layer).

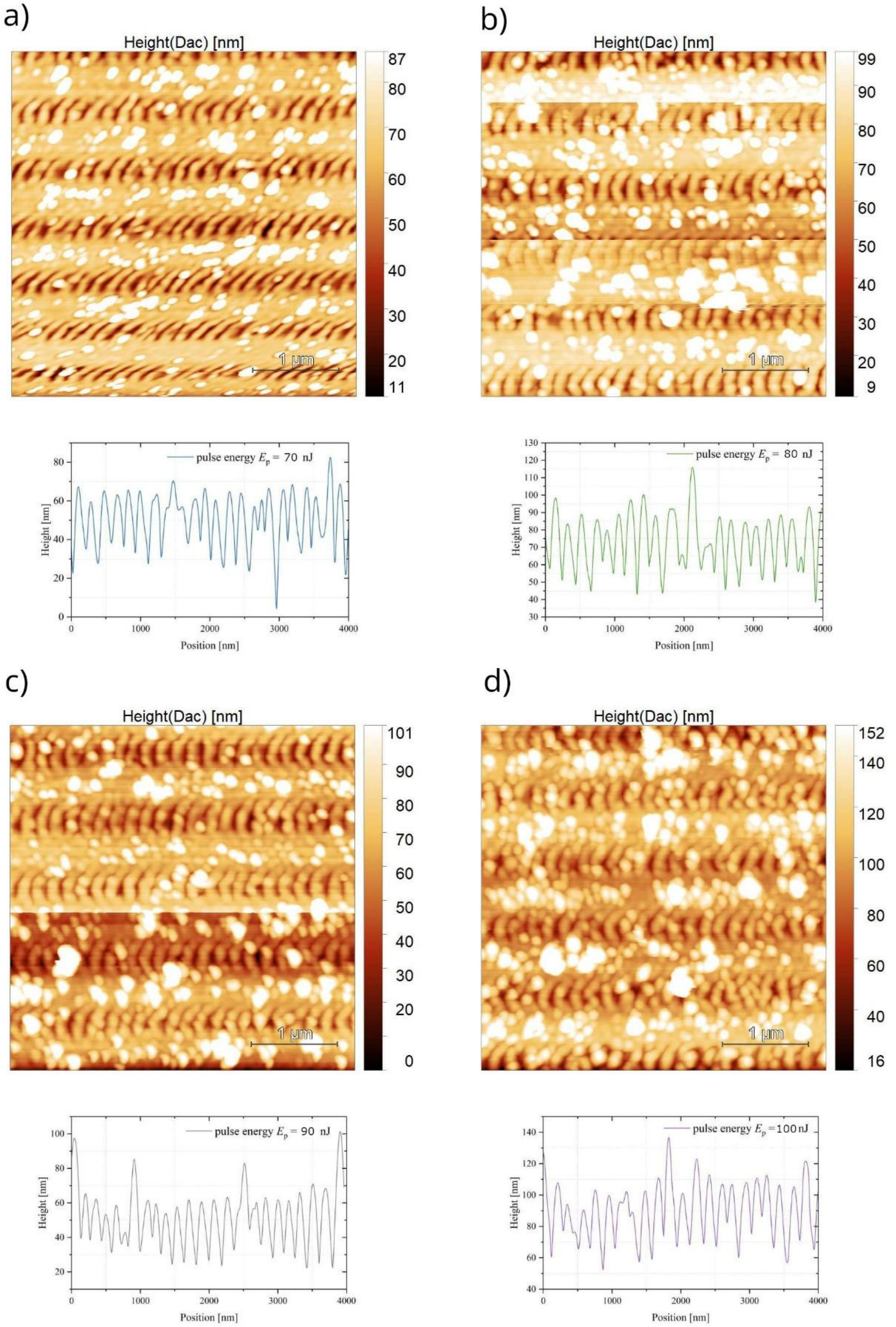

Supplementary Figure 11 | AFM pictures and the surface profiles of LIPSS structures fabricated with different laser pulse fluences, (a) 70 nJ, (b) 80 nJ, (c) 90 nJ, and (d) 100 nJ. The average depths extracted from the surface profiles are 32.5 nm, 34.8 nm, 36.0 nm, and 38.6 nm, respectively.

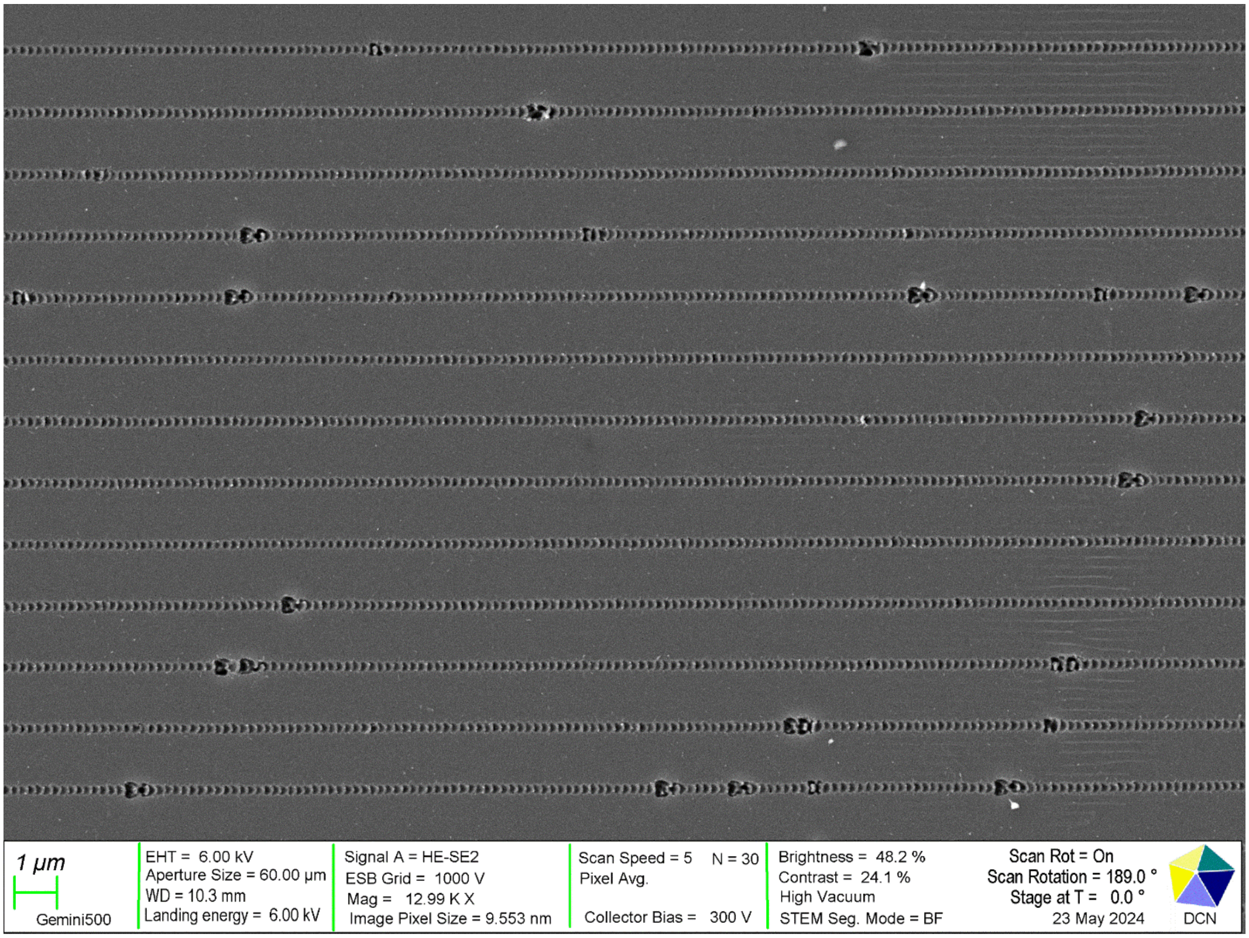

Supplementary Figure 12: Top-view SEM-scan of a set of LIPSS-gratings which were fabricated one after another with the same parameters. For the Fourier Transformation, a horizontal pixel line in the center of each LIPSS grating was chosen and then analyzed (see Figure 1d and e). The large damages in the LIPSS gratings are spots where two femtosecond-pulses instead of one reached the substrate due to randomly appearing synchronization issues between the shutter and the structuring laser.

Supplementary Note 1 – Extended analysis of the processes involved in the ripple formation

Ripple formation due to LIPSS should result in a ripple orientation perpendicular to the laser polarization direction and independent of the travel direction. However, the process of single point writing will result in ripples parallel to the travel direction and independent of the laser polarization direction. Therefore, we fabricated multiple samples consisting of horizontal and vertical lines where the same parameters were applied for corresponding pairs. In the following, we will discuss our observations and if our structures can be classified as LIPSS. The presented SEM scans show pairs of samples, where for each pair the travel direction was changed but all other parameters were kept constant. With this approach we want to examine if the ripple orientation is staying constant (independent of travel direction) and hence only depends on the laser polarization direction, or the ripple orientation is changing according to the travel direction, which will indicate the influence of the single pulse writing mechanism[3]. The laser polarization direction was always horizontal.

For pulse-to-pulse spacings below the range used for our Bragg laser resonators, we can easily demonstrate HSFL behavior (the LIPSS orientation does not change when the travel direction is changed), as shown in Supplementary Figure 13:

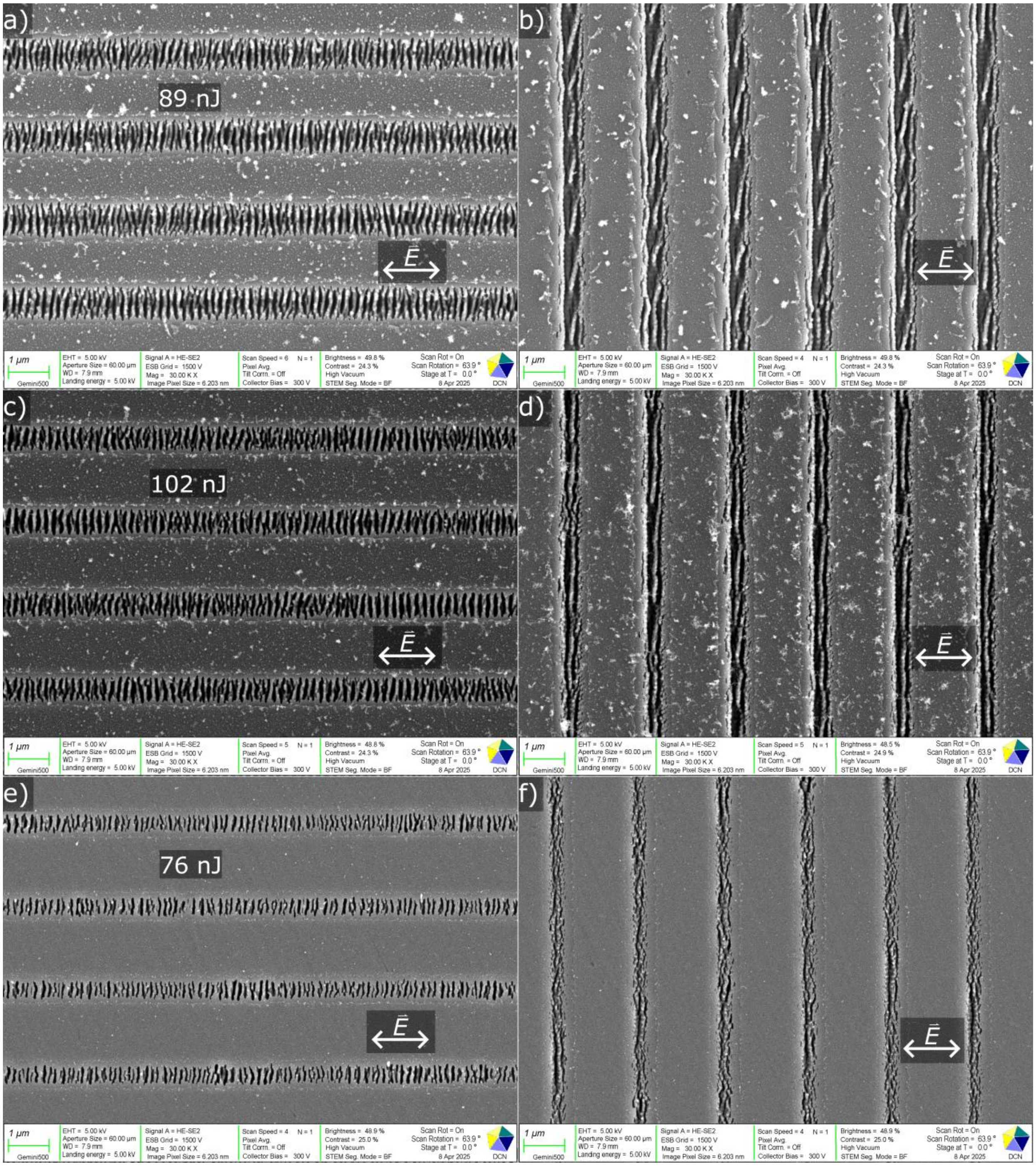

Supplementary Figure 13: LIPSS formation with small spot-to-spot spacings | The first two samples (a-d) were fabricated with a pulse-to-pulse spacing of 111 nm whereas the last sample (e-f) was fabricated with a pulse-to-pulse spacing of 143 nm. The structuring laser polarization was kept horizontal for all samples, only the travel direction was changed. The formation of HSFL can be clearly demonstrated by the orientation of the ripples being always vertical and hence perpendicular to the laser light polarization.

For a pulse-to-pulse spacing of 189 nm as used for creating lasing resonators, the clarification of the structures is not so straightforward (see Supplementary Figure 14). It can be seen that the major ripple orientation is rotating depending on the travel direction which indicates the absence of clear LIPSS behavior but rather a point-by-point writing mechanism as described by Sun *et al.*[3]. When looking at the detailed shape of the rims, small sub-ripples on the rims themselves can be observed, which consequently have an orientation perpendicular to the laser polarization direction (as expected from HSFL on glass). Additionally, there are irregularities in the structures where the main ripple orientation locally changes and is aligned perpendicular to the structuring laser polarization (as expected from LIPSS). This implies that we are in the transition zone between LIPSS formation and point-by-point writing. Due to the parallel alignment of the ripples formed by point-by-point writing and the ripples formed by LIPSS, this transition zone is not easily observable when the scanning direction is parallel to the laser polarization like during the fabrication process of our laser resonators.

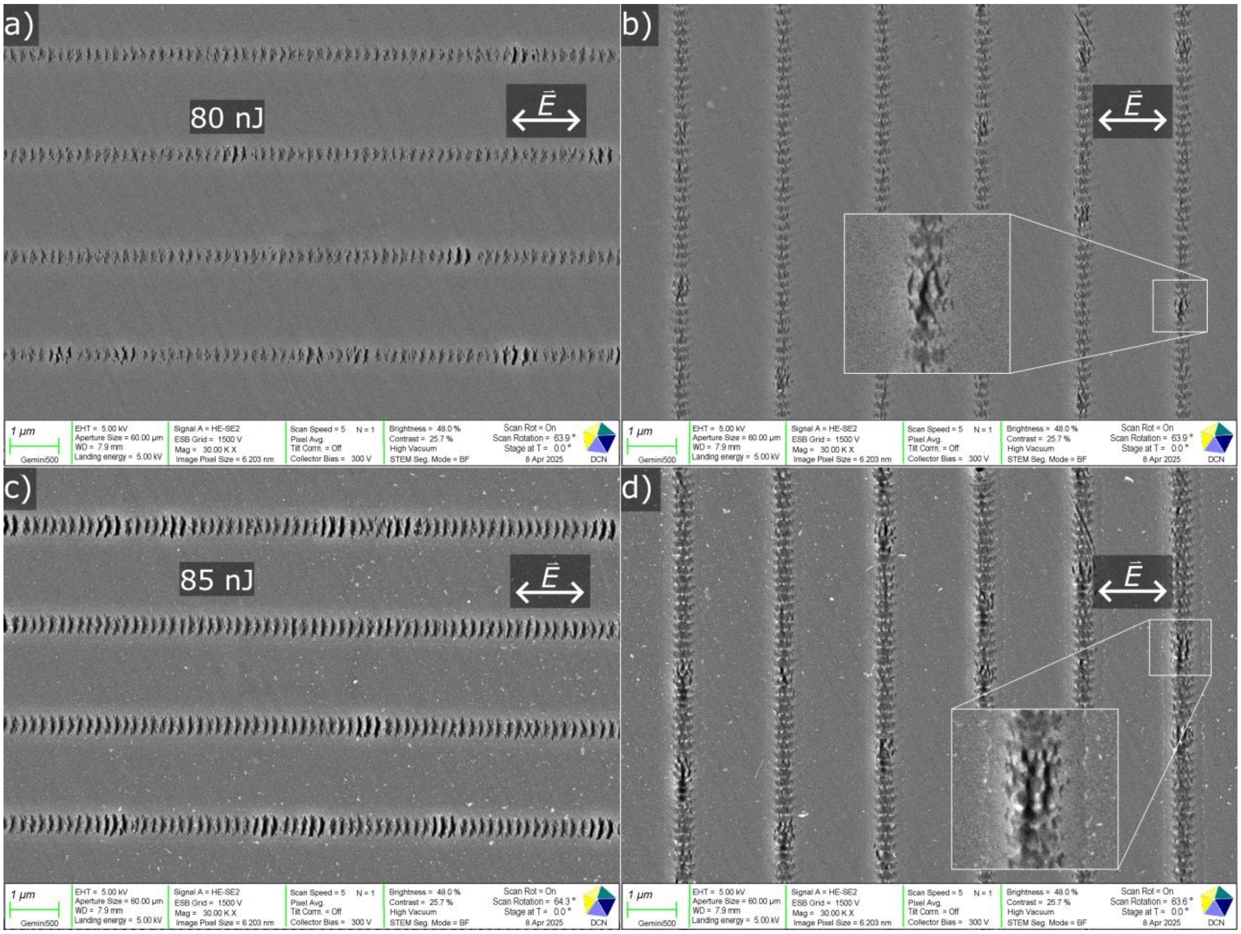

Supplementary Figure 14: Fabrication conditions similar to DFB resonator fabrication | These structures were fabricated with a pulse-to-pulse spacing of 189 nm and a pulse fluence of 80 and 85 nJ, which corresponds to the most used condition to fabricate optical gratings. The vertical lines show clear horizontal ripples, which points towards single pulse writing as the major effect involved in the ripple formation. However, closely looking at the darker defect spots, it can be seen that the ripple orientation changes locally. In these spots, LIPSS formation seems to have a higher influence on the ripple formation direction than the single pulse writing process.

Going to very low laser fluences during fabrication allows us to reach a state where the LIPSS formation is more pronounced than the point-by-point writing in the case of the travel direction being perpendicular to the laser polarization (see Supplementary Figure 15).

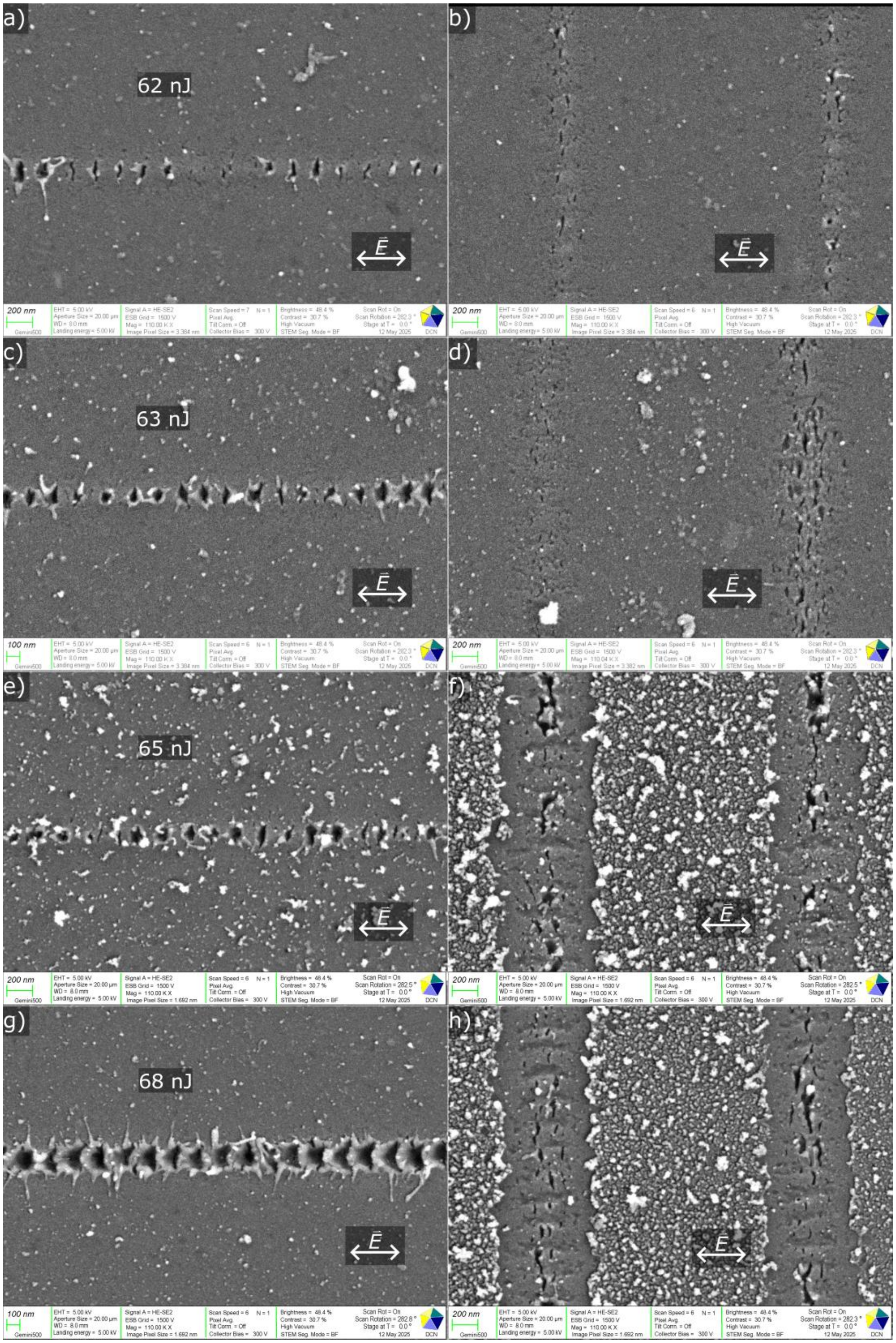

Supplementary Figure 15: LIPSS-formation at low laser pulse fluence | All structures were fabricated with a pulse-to-pulse spacing of 189 nm, like it was used for the laser resonator structures. For the lower fluence, clear vertical lines for the vertical travel direction can be seen, which clearly hints towards LIPSS

formation. With increasing laser pulse fluence, additional horizontal ripples are distinguishable, which are likely to be combing from rim formation. This indicates that with our grating periodicity of 189 nm, we are in the transition zone between LIPSS and single pulse writing, and both processes are involved in the grating formation.

To summarize, LIPSS is not always the main mechanism responsible for the ripple formation. However, we can show that LIPSS is still appearing within the range of our fabrication parameters. Since for our grating fabrication, the ripple formation direction from both mechanisms (LIPSS and single point writing) coincides, we assume that both effects play a role in the ripple formation. According to the work by Sun *et al.*[3], we are in the transition zone between Reg. II and Reg. III, which corresponds to our observations.

The exact mechanisms involved in the fabrication of the surface nanogratings are not of relevance for the application as lateral Bragg resonators for thin-film lasers. The structures fabricated with rather high pulse fluence tend to have a more sinusoidal height profile which is beneficial for the reflectivity of the grating. But when the pulse fluence is further increased and the ripples appear with notable curvature, the reflectivity is lowered and hence the observed lasing threshold higher. In that case, the phase differences between the center and the edge of the grating partly cancel out the reflected wave.

References of Supplementary Information

1. Tsutsumi, N., Nagi, S., Kinashi, K. & Sakai, W. Re-evaluation of all-plastic organic dye laser with DFB structure fabricated using photoresists. *Sci. Rep.* **6,** 34741 (2016).
2. Kozlov, V. G., Bulović, V., Burrows, P. E. & Forrest, S. R. Laser action in organic semiconductor waveguide and double-heterostructure devices. *Nature* **389,** 362–364 (1997).
3. Sun, Q., Liang, F., Vallée, R. & Chin, S. L. Nanograting formation on the surface of silica glass by scanning focused femtosecond laser pulses. *Opt. Lett.* **33,** 2713–2715 (2008).
