# Supplementary material for: Read my LIPSS: organic lasers on micromachined resonators
Source: Nat Commun. 2025 Aug 1;16:7057. doi: 10.1038/s41467-025-62502-6 (PMC12316998; doi:10.1038/s41467-025-62502-6)
Supplement: Supplementary file 2 — Reporting Summary [file 41467_2025_62502_MOESM2_ESM.pdf]

nature research

Corresponding author(s): Tiange Dong
Tobias Antrack
Karl Leo

Lasing Reporting Summary

Nature Research wishes to improve the reproducibility of the work that we publish. This form is intended for publication with all accepted papers reporting claims of lasing and provides structure for consistency and transparency in reporting. Some list items might not apply to an individual manuscript, but all fields must be completed for clarity.

For further information on Nature Research policies, including our data availability policy, see Authors & Referees.

ü Experimental design

Please check: are the following details reported in the manuscript?

1. Threshold

Plots of device output power versus pump power over a wide range of values indicating a clear threshold
- [x] Yes
- [ ] No

Main text, Fig. 3b

2. Linewidth narrowing

Plots of spectral power density for the emission at pump powers below, around, and above the lasing threshold, indicating a clear linewidth narrowing at threshold
- [x] Yes
- [ ] No

Fig. 3a, Fig. 3b

Resolution of the spectrometer used to make spectral measurements
- [x] Yes
- [ ] No

Methods

3. Coherent emission

Measurements of the coherence and/or polarization of the emission
- [ ] Yes
- [x] No

Material properties and resonator behavior are investigated. Emission is detected out-of-plane from a first-order DFB -> All collected light is scattered which randomly influences these parameters.

4. Beam spatial profile

Image and/or measurement of the spatial shape and profile of the emission, showing a well-defined beam above threshold
- [ ] Yes
- [x] No

Emission is collected out-of-plane from a first-oder DFB, all collected light is scattered from the resonator.

5. Operating conditions

Description of the laser and pumping conditions
Continuous-wave, pulsed, temperature of operation
- [x] Yes
- [ ] No

Methods, (main text)

Threshold values provided as density values (e.g. W cm^{-2} or J cm^{-2}) taking into account the area of the device
- [x] Yes
- [ ] No

Main text, Fig. 3b

6. Alternative explanations

Reasoning as to why alternative explanations have been ruled out as responsible for the emission characteristics
e.g. amplified spontaneous, directional scattering; modification of fluorescence spectrum by the cavity
- [x] Yes
- [ ] No

Main text (discussion)

7. Theoretical analysis

Theoretical analysis that ensures that the experimental values measured are realistic and reasonable
e.g. laser threshold, linewidth, cavity gain-loss, efficiency
- [x] Yes
- [ ] No

Main text (Reflectivity simulations of the resonator design)

8. Statistics

Number of devices fabricated and tested
- [ ] Yes
- [x] No

The high precision necessary to get the correct conditions results in a large effort to fabricate numerous devices.

Statistical analysis of the device performance and lifetime (time to failure)
- [ ] Yes
- [x] No

The organic gain medium degrades very fast, which makes it unsuitable for any application which relies on lifetime.
